# Dasatinib (BMS-35482) potentiates the activity of gemcitabine and docetaxel in uterine leiomyosarcoma cell lines

**DOI:** 10.1186/2053-6844-1-2

**Published:** 2014-09-30

**Authors:** Micael Lopez-Acevedo, Lisa Grace, Deanna Teoh, Regina Whitaker, David J Adams, Jingquan Jia, Andrew B Nixon, Angeles Alvarez Secord

**Affiliations:** Division of Gynecologic Oncology, Duke Cancer Institute, Durham, NC 27710 USA; Division of Gynecologic Oncology, University of Minnesota, Minneapolis, MN 55455 USA; Department of Medicine, Duke University Medical Center, Durham, NC 27710 USA; East Carolina University School of Medicine, Greenville, NC 27834 USA; DUMC 3079, Gynecologic Oncology, Duke University Medical Center, Durham, NC 27710 USA

**Keywords:** Dasatinib, Leiomyosarcoma, SRC pathway, Targeted agents, Uterine sarcoma

## Abstract

**Background:**

To explore the activity of dasatinib alone and in combination with gemcitabine and docetaxel in uterine leiomyosarcoma (uLMS) cell lines, and determine if dasatinib inhibits the SRC pathway.

**Methods:**

SK-UT-1 and SK-UT-1B uLMS cells were treated with gemcitabine, docetaxel and dasatinib individually and in combination. SRC and paxcillin protein expression were determined pre- and post-dasatinib treatment using Meso Scale Discovery (MSD) multi-array immunogenicity assay. Dose-response curves were constructed and the coefficient of drug interaction (CDI) and combination index (CI) for drug interaction calculated.

**Results:**

Activated phosphorylated levels of SRC and paxillin were decreased after treatment with dasatinib in both cell lines (p < 0.001). The addition of a minimally active concentration of dasatinib (IC_25_) decreased the IC_50_ of each cytotoxic agent by 2-4 fold. The combination of gemcitabine-docetaxel yielded a synergistic effect in SK-UT-1 (CI = 0.59) and an antagonistic effect in SK-UT-1B (CI = 1.36). Dasatinib combined with gemcitabine or docetaxel revealed a synergistic anti-tumor effect (CDI < 1) in both cell lines. The triple drug combination and sequencing revealed conflicting results with a synergistic effect in SK-UT-1B and antagonistic in SK-UT-1.

**Conclusion:**

Dasatinib inhibits the *SRC* pathway and yields a synergistic effect with the two-drug combination with either gemcitabine or docetaxel. The value of adding dasatinib to gemcitabine and docetaxel in a triple drug combination is uncertain, but may be beneficial in select uLMS cell lines. Based on our pre-clinical data and known activity of gemcitabine and docetaxel, further evaluation of dasatinib in combination with these agents for the treatment of uLMS is warranted.

**Electronic supplementary material:**

The online version of this article (doi:10.1186/2053-6844-1-2) contains supplementary material, which is available to authorized users.

## Background

Leiomyosarcomas (LMS) are a rare and aggressive type of uterine malignancy that has an extremely poor prognosis. Uterine sarcomas represent only 3-9% of all uterine malignancies and LMS account for 40% of all uterine sarcomas [1, 2]. Even in the setting of early-stage disease 53 to 71% of women will develop recurrences that are often extra-pelvic and incurable. The prognosis is dismal with a historical progression-free survival rate at 2 years (PFS_2 yrs_) of only 30% for patients treated with surgery alone [3].

Chemotherapy with a combination of gemcitabine and docetaxel has shown the most promise to date. The overall response rate has ranged from 42% to 53% with a median duration of response of greater than 7 months in women with unresectable LMS [4]. Most recently, adjuvant gemcitabine and docetaxel was evaluated in women with completely resected stage I to IV uterine LMS. The PFS_2 yrs_ was 45% with a median PFS of 13 months and the median survival was not yet reached [3]. Despite these modest improvements, there is an urgent need for innovative therapeutic approaches.

One novel and promising therapeutic agent is dasatinib (BMS-354825) (NSC 732517). Dasatinib is a potent, orally-bioavailable, small molecule inhibitor that has been shown to inhibit at least five protein tyrosine kinases/kinase families: SRC family kinases, BCR-ABL, c-KIT, EPHA2 and the PDGFβ receptor [5, 6]. SRC kinase interacts with a variety of receptor tyrosine kinases such as EGFR, PDGFR, and c-KIT as well as other cellular factors such as focal adhesion kinase (FAK). These pathways are integral components of cellular function and regulate cellular migration, proliferation, survival, angiogenesis, and metastasis. The SRC pathway and its substrates (FAK) as well as c-KIT, EGFR and PDGF tyrosine kinase receptors have been found to be overexpressed in a wide variety of sarcomas including LMS [7–9]. Most recently, Shank *et al*. showed that EGFR, VEGF and c-KIT is expressed in uterine LMS specimens [9]. In particular, 57% of uterine LMS specimens express c-KIT.

Given the activity of gemcitabine and docetaxel as well as the pre-clinical data regarding the SRC pathway in LMS, we sought to explore the activity of dasatinib (a SRC inhibitor) in combination with these cytotoxic agents in uLMS.

## Methods

### Drugs

Gemcitabine and docetaxel were purchased from Sigma (St. Louis, MO). Dasatinib (BMS-354825) was provided by Bristol-Myers-Squibb (Princeton, NJ) *via* the National Cancer Institute (NCI). Dasatinib, gemcitabine and docetaxel were dissolved in dimethylsulfoxide (DMSO). Concentrated stock solutions of all drugs were stored at -25°C.

### Cell culture

SK-UT-1 and SK-UT-1B cell lines were obtained from the American Type Culture Collection (Manassas, VA). SK-UT-1 and SK-UT-1B cell lines are uterine in origin. Cell lines were grown and maintained in monolayer culture in RPMI 1640 (Gibco) media supplemented with 10% fetal bovine serum (Hyclone, Logan UT), 1% sodium pyruvate, 100 units/ml penicillin, 100 ug/ml stremptomycin and 1% nonessential amino acids in a humidified chamber containing 5% CO_2_.

### Meso scale discovery (MSD) analysis

MSD analysis was performed for SRC and paxillin. Paxillin is a SRC pathway substrate that is phosphorylated by SRC and FAK upon integrin binding or growth factor stimulation. Cells were seeded at 3×10^6^ cells per plate of each cell line, and allowed to reach confluence over 24 hours. Cells were incubated at 37°C for 24 hours with dasatinib at a escalating concentrations of 30, 100 and 500 nmol/L. Controls were treated with DMSO. Anti-total Src antibody (tSrc) (Cell Signaling Technology, Inc., Danvers, MA, Cat#2108), anti-pSrc pY418 antibody (Invitrogen, Carlsbad, CA, Cat# 44660G), anti-total paxillin antibody (Cell Signaling Technology, Inc., Danvers, MA, Cat#2542), or anti-phospho paxillin (Tyr118) antibody (Cell Signaling Technology, Inc., Danvers, MA, Cat#2541) were added at 1ug/ml to bare, goat anti-mouse plates (MSD, Gaithersburg MD), and incubated at room temperature (R.T.) for 1 hour. The plates were washed with TBS/ 0.05% Tween-20 three times and protein lysate from SK-UT-1 (20 ug total protein) or SK-UT-1B (20 ug total protein) cells were added and incubated for 2 hours at R.T. Sulfo-TAG (MSD, Cat#R91AN-1) labeled anti-Src antibody (R&D, Cat# AF3389) were then added to the plates and incubated for 1 hour at room temperature after plates were washed. The plates were imaged and analyzed using a MSD Sector Imager 2400 and associated software. The electroluminescence value was normalized to each control and plotted as a percent of control. Statistical analysis was performed using two-tailed unpaired *t*-test.

### Cell proliferation assay

Tumor cells were seeded at a density of 2,500 cells/well in a 96-well plate, and allowed to reach 70% confluence over 24 hours. Cells were then incubated with each drug at 37°C for 72 hours with escalating doses: docetaxel (0.1 nmol/L-1000 nmol/L), gemcitabine (0.1 nmol/L-100 nmol/L) and dasatinib (10 nmol-4000 nmol/L). Control wells contained RPMI media only. All experiments were done using exponentially proliferating cells. After the 72 hours drug incubation period, 5 μl of ATP luminescence solution was added to each well and cell proliferation was measured by ATP content using the Luminescence ATP cell detection assay system according to manufacturer’s recommendations. Experiments to determine the IC_50_ were performed in triplicate.

The percentage of growth inhibition was calculated using the following: survival ratio = # live cells_treated group_ /#live cells_control group_ × 100. The half maximal inhibitory concentration (IC_50_) was defined as the drug concentration at which the 50% of the cell growth was inhibited and was analyzed using GraphPad Prism software (version 4.03 San Diego, CA). Single-agent dose response curves were constructed and the IC_50_ for gemcitabine and docetaxel was computed from the best fitting transition functions (determined by F-statistic). The average IC_50_ of all experiments performed was chosen as the final IC_50_. Given the lack of significant activity of dasatinib as a single-agent, the IC_25_ was calculated from the dose response curve. The cells were subsequently treated with combinations of gemcitabine and docetaxel; dasatinib and gemcitabine; dasatinib and docetaxel; and the three-drug simultaneous and sequential combination of gemcitabine, docetaxel, and dasatinib. For gemcitabine and docetaxel, fixed-ratio molar concentrations ranging from 0.125 to 4 multiples of the single-drug IC_50_ was used. Dasatinib was added at a fixed 1:1 ratio using the IC_25_.

### Two-drug combination effect evaluation and statistical methodologies

The IC_50_ obtained for single-agent gemcitabine and docetaxel was compared to the IC_50_ calculated for each cytotoxic agent after adding dasatinib IC_25_. To analyze the drug interaction between both cytotoxic agents and dasatinib combined with either agent, the coefficient of drug interaction (CDI) was calculated. CDI is defined by the following formula; CDI = AB/(A × B) [10, 11]. According to the absorbance of the luminescence of each group, AB is the ratio of the two-drug combination group to the control group, and A or B is the ratio of the single drug group to the control group. CDI < 1 indicates synergism, CDI < 0.7 significant synergism, CDI = 1 additivity and CD > 1 antagonism [11]. Due to the lack of significant activity of dasatinib and the inability to obtain an IC_50_ for this agent, the CDI formula was utilized to evaluate the anti-proliferative effect between dasatinib and each cytotoxic agent. This formula permits calculation of drug-to-drug interaction without a requisite IC_50_. All experiments were performed in duplicate.

### Triple drug simultaneous and sequential combination effect evaluation and statistical methodologies

To evaluate the growth inhibition effect of dasatinib with gemcitabine and docetaxel in a triple-drug combination, we used the median effect method, which takes into account the potency of each drug combination and the shape of the dose-response curve [12, 13]. Composite dose response curves were obtained from three independent experiments and the median effective dose, Dm (equivalent to the IC_50_) was computed using CalcuSyn software (Biosoft, Cambridge, UK). Drug interaction was assessed by the combination index (CI) method of Chou and Talalay [12, 13]:

$$\begin{array}{ll} \mathrm{CI}\;= & \left(D\right)A\;/\left(\mathrm{Dx}\right)A\;+\;\left(D\right)B\;/\left(\mathrm{Dx}\right)B\;\\ +\;\alpha \left(D\right)A\;\left(D\right)B\;/\left(\mathrm{Dx}\right)A\left(\mathrm{Dx}\right)B \end{array}$$

where D is the dose that yields x% growth inhibition and α = 0 for mutually exclusive drugs (the drugs have similar sites of action). Combination index scale was defined as: CI <0.9 synergistic, CI = 0.9-1.1 additive, CI = 1.1-1.2 slight antagonism, CI = 1.2-1.45 moderate antagonism, CI = 1.45-3.3 antagonism, CI = 3.3-10 strong antagonism.

In contrast to the two-drug combination where the CDI formula was used, the median effect (CI formula) method was utilized to evaluate triple-drug interaction. This was possible because both gemcitabine and docetaxel reached an IC_50_ as single agents. For sequencing studies, drug exposures were separated by 24 hours. The combination index between gemcitabine and docetaxel was computed with and without the addition of dasatinib IC_25_. All experiments were performed in triplicate.

## Results

### Meso scale discovery (MSD) analysis

#### SRC protein expression

**Figure 1:**
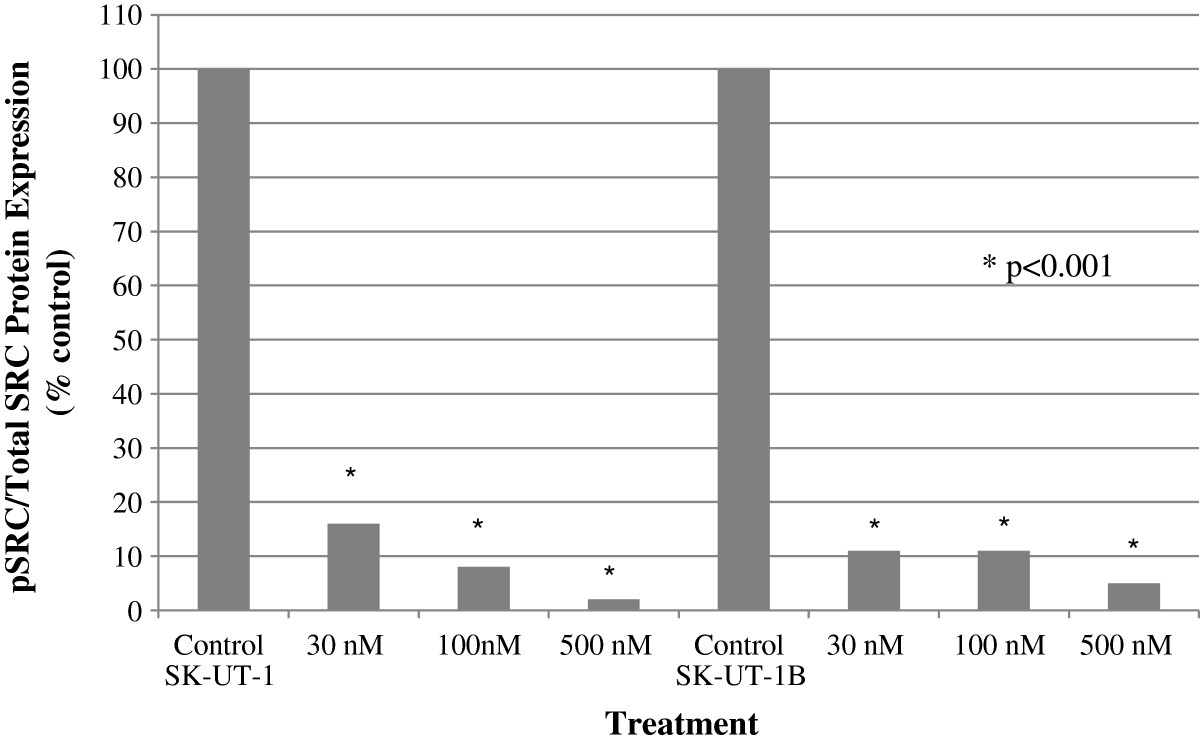
**The pSRC/tSRC ratio after treatment with single-agent dasatinib.** In SK-UT-1, activation of the SRC kinase (pSRC/tSRC) pathway was inhibited by dasatinib at 30 nm (84%), 100 nm (92%) and 500 nm (98%). In SK-UT-1B, the SRC kinase activity was reduced in the presence of dasatinib at 30 nm (91%), 100 nm (91%) and 500 nm (95%).

The level of SRC activation (pSRC) was determined using MSD analysis. In SK-UT-1, treatment with dasatinib resulted in a loss of SRC activation (pSRC/tSRC) at 30 nm (16%; p < 0.001), 100 nm (8%; p < 0.001) and 500 nm (2%; p < 0.001) (Figure 1). The pSRC signal from SK-UT-1 was at least 15-fold higher than the pSRC signal from the control cells (Additional file 1: Figure S1). In SK-UT-1, there was an increase in tSRC after treatment with single-agent dasatinib at 30 nm (148%, p < 0.001), 100 nm (181%, p < 0001) and 500 nm (172%, p < 0.001) compared to controls (Additional file 2: Figure S2). In contrast, pSRC levels were significantly decreased after treatment with dasatinib at 30 nm (24%, p < 0.001), 100 nm (14%, p < 0.001) and 500 nm (3%, p < 0.001) (Additional file 3: Figure S3).

In SK-UT-1B, there was a loss of SRC activation at 30 nm (11%, p < 0.001), 100 nm (11%, p < 0.001) and 500 nm (5%, p < 0.001) compared to controls (Figure 1). There was a decrease in pSRC levels after treatment with single-agent dasatinib at 30 nm (17%, p < 0.001), 100 nm (7%, p < 0.001) and 500 nm (4%, p < 0.001) and an increase in tSRC after treatment with single-agent dasatinib at 30 nm (152%, p < 0.001), but a decrease at 100 nm (64%, p < 0.001) and 500 nm (74%, p < 0.001) (Additional file 2: Figure S2 and Additional file 3: Figure S3).

#### Paxillin protein expression

**Figure 2:**
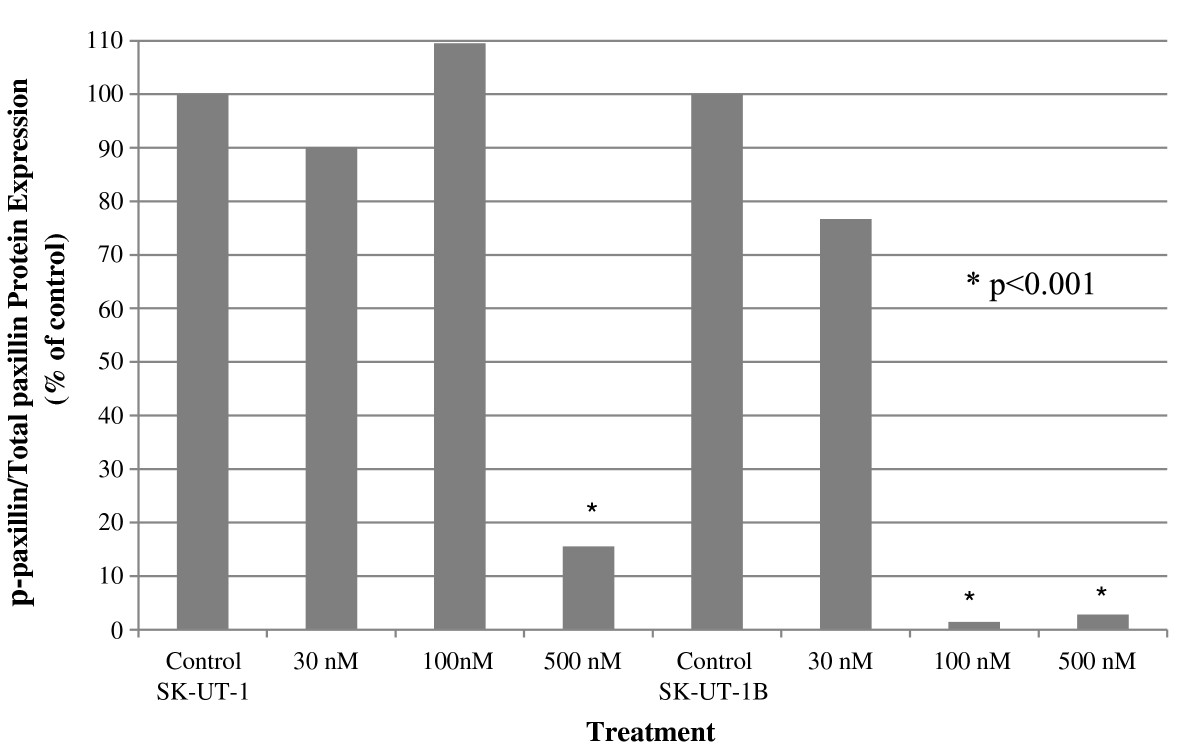
**The p-paxillin/t-paxillin ratio after treatment with single-agent dasatinib.** Activation of paxillin (p-paxillin/t-paxillin) was inhibited by the presence of dasatinib at 500 nm in SK-UT-1 (16%); and 30 nm (14%), 100 nm (1%) and 500 nm (3%) in SK-UT-1B.

In SK-UT-1, the ratio of p-paxillin/t-paxillin was unchanged after treatment with dasatinib at 30 nm (90%, p < 0.001) and 100 nm (110%, p < 0.001), but there was a significant reduction at 500 nm (16%, p < 0.001) (Figure 2). There was a significant loss of t-paxillin expression after treatment with dasatinib at 500 nm (73%, p = 0.01), but no change at 30 nm (113%, p = 0.06) and 100 nm (92%, p = 0.2) compared to controls (data not shown). Similar results were observed for p-paxillin levels, with a significant loss after treatment with dasatinib at 500 nm (11%, p < 0.001), but no change at 30 nm (100%) or 100 nm (100%) (data not shown).In SK-UT-1B, activation of paxillin (p-paxillin/t-paxillin) was significantly inhibited by the presence of dasatinib at 30 nm (76%), 100 nm (1%) and 500 nm (3%) (Figure 2). There was an increase in t-paxillin expression after treatment with dasatinib at 100 nm (198%, p < 0.001), but no change at 30 nm (76%, p = 0.02) or 500 nm (93%, p = 0.2) (data not shown). The expression of p-paxillin was reduced by 42% after treatment with dasatinib at 30 nm (p < 0.001) and by 97% (p < 0.001) at 100 and 500 nm (data not shown).

### Anti-proliferative activity of single-agent gemcitabine, docetaxel and dasatinib

**Figure 3:**
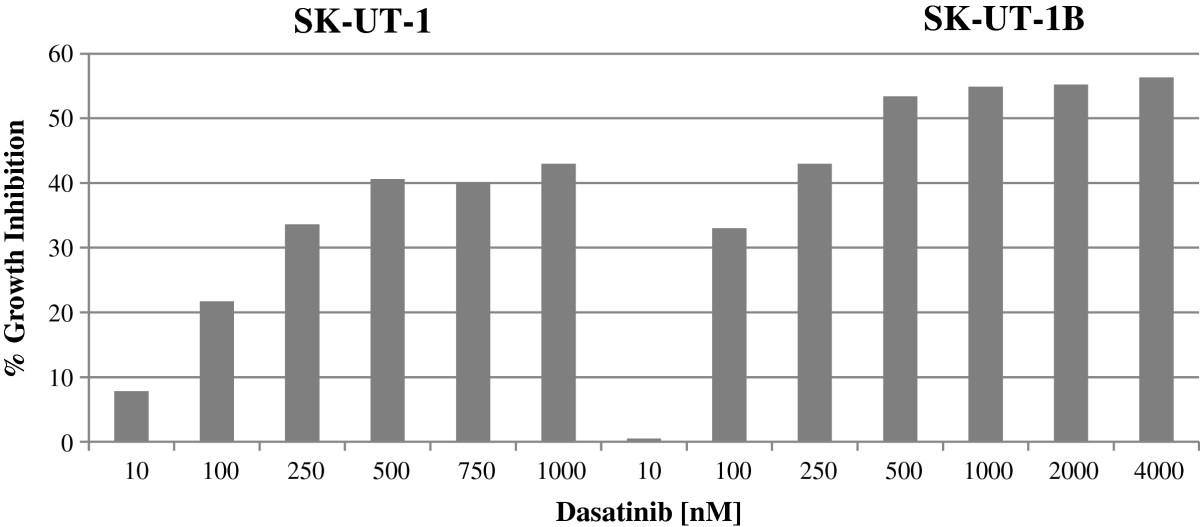
**Growth inhibition assay.** Dasatinib showed minimal to modest activity against leiomyosarcoma cell lines. Maximal growth inhibitory effect for SK-UT-1 and SK-UT-1B was 42.8% and 55% respectively.

The IC_50_ for gemcitabine and docetaxel as single agents was calculated for each cell line (Table 1). Growth inhibition with dasatinib was first detected at 100 nmol for SK-UT-1 (21.7% inhibition) and for SK-UT-1B (32.8%). Using increasing concentrations of dasatinib, the maximal growth inhibitory effect of dasatinib for SK-UT-1 was 42.8% (1000 nm) and 55.5% (4000 nm) for SK-UT-1B (Figure 3). The dasatinib IC_50_ for SK-UT-1B was 381 nmol/L. Higher concentrations did not achieve greater growth inhibition. In the SK-UT-1 cell line an IC_50_ was not reached. The IC_25_ (25% growth inhibition) was therefore calculated from dose response curves for each cell line. The IC_25_ for dasatinib was 100 nm for both cell lines.

**Table 1:** **IC**_**50**_ **of each cytotoxic agent alone and in combination with dasatinib (IC**_**25**_ **) and coefficient of drug interaction (CDI)**

Cell Line	Drugs	IC _50_	CDI
**SK-UT-1**			
	Gemcitabine	4.02 (SD ± 1.0)	
	Docetaxel	4.30 (SD ± 0.5)	
	Gemcitabine:Dasatinib IC_25_	2.35 (SD ± 1.4)	0.72
	Docetaxel:Dasatinib IC_25_	1.15 (SD ± 0.07)	0.80
**SK-UT-1B**			
	Gemcitabine	2.97 (SD ± 0.5)	
	Docetaxel	0.94 (SD ± 0.6)	
	Gemcitabine:Dasatinib IC_25_	0.85 (SD ± 0.6)	0.83
	Docetaxel:Dasatinib IC_25_	0.52 (SD ± 0.4)	0.93

SD = standard deviation.

IC_50_ = 50% of maximal inhibitory concentration.

IC_25_ = 25% of maximal inhibitory concentration.

CDI = Coefficient of drug interaction. Calculation utilize when one drug does not achieve an IC_50._

CDI scale = CDI < 1 indicates synergism between dasatinib and cytotoxic agents, whereas CDI < 0.7 indicates significant synergism.

### Drug interaction assessment for combination dasatinib and cytotoxic chemotherapy

**Figure 4:**
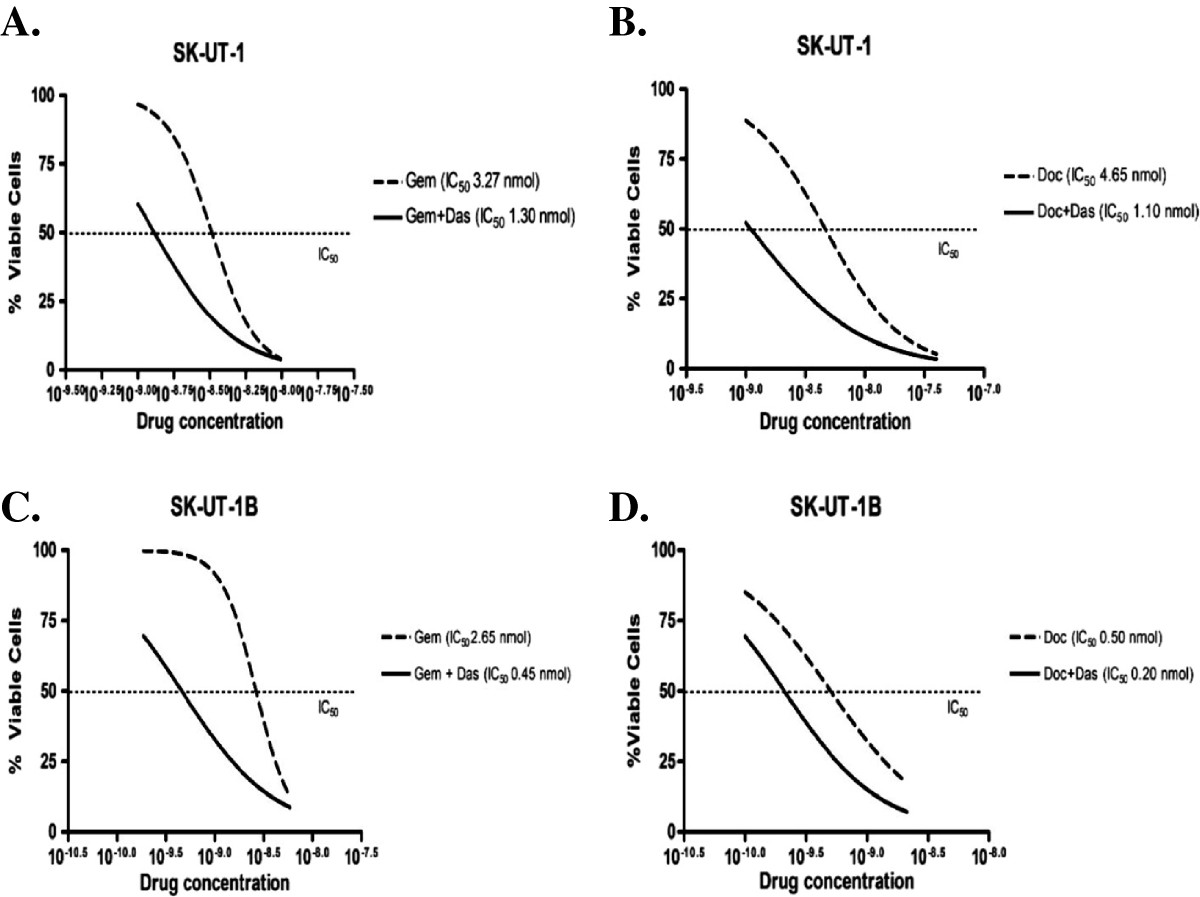
**IC**_**50**_ **obtained from single agent response curves for gemcitabine and docetaxel was compared to the IC**_**50**_ **obtained after adding dasatinib at a minimally active concentration (IC**_**25**_ **).** Graphics above represents the results of a single experiment for SK-UT-1 **(A & B)** and SK-UT-1B **(C & D)**. (Gem = gemcitabine; Das = dasatinib; Doc = docetaxel).

**Figure 5:**
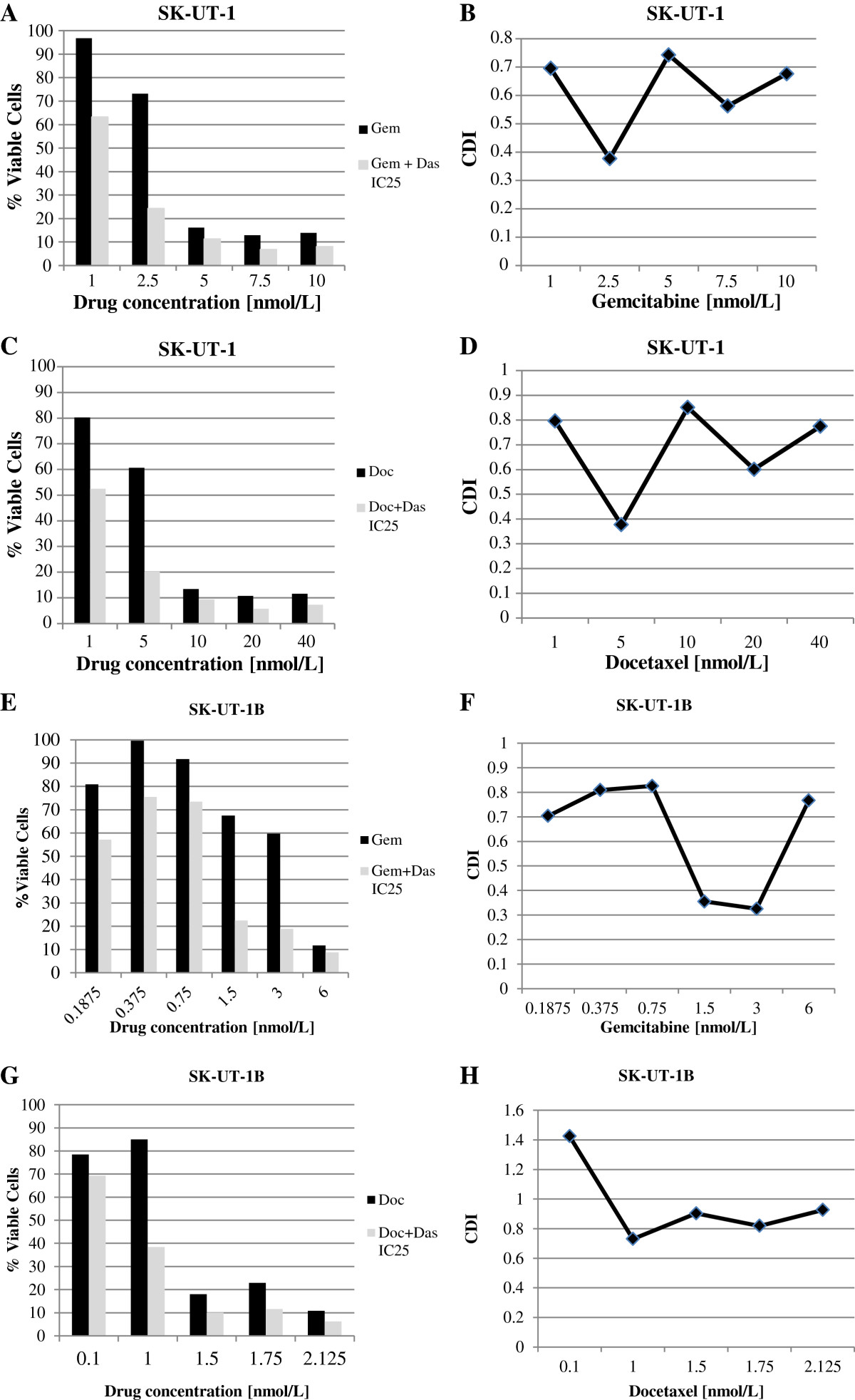
**The inhibitory effect of gemcitabine and docetaxel at different concentrations combined with dasatinib IC**_**25**_ **and the coefficient of drug interaction (CDI).** CDI < 1 indicates synergism, CDI < 0.7 significant synergism, CDI = 1 additivity and CD > 1 antagonism. In SK-UT-1, the combination of gemcitabine-dasatinib, at all concentrations analyzed demonstrated decreased cell viability **(A)** and yielded a CDI <1 indicating synergistic effects (average CDI = 0.72) **(B)**; the combination of docetaxel-dasatinib demonstrated decreased cell viability **(C)** and synergistic effects (average CDI = 0.80) **(D)**. In SK-UT-1B the combination of gemcitabine-dasatinib, at all concentrations analyzed demonstrated decreased cell viability **(E)** and yielded a CDI <1 indicating synergistic effects (average CDI = 0.83) **(F)**; the combination of docetaxel-dasatinib demonstrated decreased cell viability **(G)** and synergistic effects (CDI = 0.93) **(H)**.

The IC_25_ of dasatinib calculated for each cell line was used in combination with different concentrations of gemcitabine (e.g. 10, 7.5, 5, 2.5, 1 nm) and docetaxel (e.g. 40, 20, 10, 5, 1 nm) mixed at a fixed ratio (1:1), respectively. The IC_50_ obtained from the single agent response curves for both gemcitabine and docetaxel was then compared to the IC_50_ calculated after adding dasatinib (IC_25_). Results showed that the minimally active dose of dasatinib reduced the IC_50_ of both gemcitabine and docetaxel for each cell line ranging from 2:1 to 4:1 (2-4 fold inhibition) (Table 1) (Figure 4).For SK-UT-1 and SK-UT-1B, the combination of gemcitabine-dasatinib at all concentrations analyzed demonstrated decreased cell viability (Figure 5A and E) and yielded a CDI <1 indicating synergistic effects (average CDI = 0.72 and 0.83 for SK-UT-1 and SK-UT-1B, respectively) (Figure 5B and F). Similarly, in both cell lines the combination of docetaxel-dasatinib demonstrated decreased cell viability (Figure 5C and G) and synergistic effects (average CDI = 0.80 and 0.93 for SK-UT-1 and SK-UT-1B, respectively) (Figure 5D and H).

The median effect analysis for combining gemcitabine and docetaxel revealed synergistic effect in SK-UT-1 (CI = 0.59) and moderate antagonism in SK-UT-1B (CI = 1.36) (Table 2). The results of the simultaneous and sequential drug experiments using dasatinib IC_25_, gemcitabine and docetaxel in a triple-drug combination are shown in Table 2. The greatest anti-proliferative activity (synergistic) in SK-UT-1 was seen with the simultaneous triple-drug combination of dasatinib, gemcitabine and docetaxel (CI = 0.46). The remainder of the sequential triple-drug combinations revealed a moderate antagonistic effect (CI = 1.07-1.8) (Table 2). In SK-UT-1B, the simultaneous triple-drug combination of gemcitabine, docetaxel and dasatinib revealed moderate antagonistic effect (anti-proliferative effect (CI = 1.4)). Sequential dasatinib followed by combination gemcitabine and docetaxel as well as gemcitabine followed by combination docetaxel and dasatinib yielded synergistic effects (CI = 0.9 for both). In contrast, combination dasatinib and docetaxel followed by gemcitabine demonstrated an additive effect (CI = 1.0). The remaining sequential combinations produced an antagonistic effect (CI ranging from 1.21-1.5) (Table 2).

**Table 2:** **The combination index (CI) in uterine LMS cell lines**

Cell Line	Drugs	Combination index (CI) at the IC _50_
**SK-UT-1**		
	Gemcitabine:Docetaxel	0.59
	Gemcitabine + Docetaxel + Dasatinib	0.46
	Dasatinib- > Gemcitabine + Docetaxel	1.77
	Gemcitabine + Docetaxel- > Dasatinib	1.14
	Dasatinib + Gemcitabine- > Docetaxel	1.32
	Dasatinib + Docetaxel- > Gemcitabine	1.07
	Gemcitabine- > Docetaxel + Dasatinib	1.8
	Docetaxel- > Gemcitabine + Dasatinib	N/D
**SK-UT-1B**		
	Gemcitabine:Docetaxel	1.36
	Gemcitabine + Docetaxel + Dasatinib	1.4
	Dasatinib- > Gemcitabine + Docetaxel	0.9
	Gemcitabine + Docetaxel- > Dasatinib	1.36
	Dasatinib + Gemcitabine- > Docetaxel	1.21
	Dasatinib + Docetaxel- > Gemcitabine	1.0
	Gemcitabine- > Docetaxel + Dasatinib	0.9
	Docetaxel- > Gemcitabine + Dasatinib	1.5

Combination index scale: CI <0.9 synergistic, CI = 0.9-1.1 additive, CI = 1.1-1.2 slight antagonism,

CI = 1.2-1.45 moderate antagonism, CI = 1.45-3.3 antagonism, CI = 3.3-10 strong antagonism.

N/D = not determined.

## Discussion and conclusion

Dasatinib has not been shown to be a potent agent when used alone in a variety of solid tumors [14]. Schrage *et al*. previously reported their findings of dasatinib in chondrosarcoma cell lines [15]. The maximum percent of growth inhibition in the chondrosarcoma cell lines was approximately 50%. They also explored dasatinib’s effect on SRC phosphorylation and caspase-3- mediated apoptosis. Although dasatinib treatment of chondrosarcoma decreased SRC phosphorylation, indicating target inhibition, it did not result in an increase in apoptosis. Therefore, the inhibition of SRC and its family of kinases was not sufficient to promote cell death. In a large clinical trial of patients with incurable sarcoma, which included 47 participants with LMS, dasatinib did not have a significant anti-tumor activity as a single agent [16]. Based on pre-clinical and clinical data, it appears that single agent dasatinib does not have significant clinical activity in soft tissue sarcomas.

However, our pre-clinical study investigating the activity of dasatinib in combination with gemcitabine and docetaxel in uterine LMS demonstrates that dasatinib acts synergistically with gemcitabine or docetaxel in a two-drug combination and in select triplet combinations in the analyzed uterine LMS cell lines. Our findings in sequential and triple combination yielded conflicting results. Interestingly, the simultaneous triple-drug combination of dasatinib IC_25_, gemcitabine and docetaxel in SK-UT-1 yielded a synergistic effect, although the magnitude of that effect is probably minimal (CI = 0.59 to 0.46). Conversely, the gemcitabine and docetaxel doublet demonstrated an antagonistic effect in SK-UT-1B cells and that effect was not reversed with the addition of dasabinib in the simultaneous triple-drug combination. However, in select sequencing experiments, dasatinib enhanced the anti-proliferative effect of gemcitabine and docetaxel; gemcitabine + docetaxel + dasatinib; dasatinib followed by docetaxel + gemcitabine; and gemcitabine followed by docetaxel + dasatinib. In the latter two experiments, this finding may be due to avoidance of the antagonistic interaction of the gemcitabine and docetaxel doublet in the SK-UT-1B cell line as well as partially attributed to the synergistic effect of the dasatinib doublet. The results with the sequential dasatinib followed by combination gemcitabine and docetaxel indicate a possible role for priming with dasatinib.

We hypothesize that our synergistic findings may in part be due to the inhibition of the SRC pathway. Our MSD analysis revealed that dasatinib inhibited the SRC pathway based on reduction of pSRC and p-paxillin protein expression and ratios pSRC/tSRC and p-paxcillin/t-paxillin. Even at very low doses of dasatinib (30 nM), the SRC pathway was inhibited in both uLMS cell lines. This suggests that an optimal biologic effect on SRC can occur with low doses of dasatinib. However, expression of paxillin was only inhibited at high doses. A possible explanation for this is that downstream substrates of SRC pathway may be activated via different pathways. The SRC pathway is a complex pathway with convergent and divergent interactions. It is not clear that the anti-proliferative effect we noted in our study was a direct result of inhibition of the SRC pathway. Most recently, Shank *et al*. showed that EGFR, VEGF and especially c-KIT were expressed in uterine LMS specimens [9]. In particular, 57% of uterine LMS specimens expressed c-KIT. Therefore, dasatinib’s anti-proliferative effect seen in our study may be via the inhibition of the c-KIT pathway in addition to or instead of inhibition of the SRC pathway.

Another important property of dasatinib not related to cytotoxicity, lies in its ability to inhibit migration and cell invasion. Dasatinib has been shown to inhibit cell migration in non-small cell lung cancer, head and neck squamous cell cancer, neuroblastoma, and multiple soft tissue and bone sarcoma cell lines [8, 17, 18]. Johnson and colleagues reported that the anti-migratory effects of dasatinib were present regardless of the effects seen on proliferation and survival [17]. In addition, Shor *et al*. showed that dasatinib significantly inhibited cellular invasion using matrigel invasion chambers. With the exception of one rhabdomyosarcoma cell line, the IC_50_ dose to inhibit cell migration and invasion in the soft tissue sarcoma cell lines ranged from 4-65 nmol/L and was similar to the IC_50_ dose (range 3 nmol/L to 68 nmol/L) to inhibit SRC phosphorylation [8]. This finding suggests that SRC inhibition may be responsible for suppression of sarcoma cellular migration and invasion. The universal effects seen on migration and invasion suggest that beneficial clinical effects may be achieved without direct cancer cell cytotoxicity and provides a possible role of dasatinib in preventing metastasis. Inhibition of metastasis is especially important in aggressive tumors such as LMS which are known to rapidly spread to distant sites.

While we and others have reported synergistic and additive effects with dasatinib and a variety of chemotherapeutic agents in preclinical studies of ovarian and breast cancer cell lines and ovarian cancer xenografts [19–21], the clinical activity of dasatinib either alone or in combination with cytotoxic agents in solid tumors has been disappointing. In a phase I trial of dasatinib in combination with paclitaxel and carboplatin in patients with advanced or recurrent ovarian cancer, the triplet combination demonstrated clinical activity but was also associated with a higher than expected rate of hematologic toxicity. Pharmacokinetic analysis showed that concurrent administration of dasatinib with paclitaxel did not significantly alter either dasatinib or paclitaxel drug concentrations [14]. Two randomized studies of another SRC inhibitor, saracatinib, with chemotherapy in ovarian cancer demonstrated no improvement in response rate or survival, but higher toxicity [22, 23]. Therefore, the addition of dasatinib to chemotherapy is unlikely to be beneficial in an unselected patient population and there is a need to identify biomarkers that can be used to direct therapy. Of note, these studies used the maximum tolerated dose (MTD). In our data, we observed an increased antiproliferative effect of dasatinib in combination with cytotoxic agents at a minimal active dose. Hence, combination effects may be beneficial at lower doses of dasatinib in the treatment of uterine leiomyosarcoma.

Up-regulation of the *SRC* pathway has been shown to be associated with resistance to cytotoxic therapy. In mucinous ovarian cancer cell lines, Matsuo *et al*. revealed that treatment with oxaliplatin induced phosphorylation of SRC kinase and this contributed to the chemoresistance observed in this tumor. This induced activity was subsequently inhibited by concurrent administration of dasatinib, which resulted in a synergistic anti-tumor effect [24].

To our knowledge, there are no clinical trials that have evaluated the toxicity of gemcitabine and docetaxel in combination with dasatinib. In a prospective trial of women with completely resected uterine leiomyosarcoma, treatment with gemcitabine plus docetaxel was generally well-tolerated. Potential serious toxicities associated with gemcitabine plus docetaxel in the advanced disease setting included pulmonary toxicity and myelosuppression requiring administration of granulocyte-colony stimulating factor [3]. Based on our prior experience, we anticipate that the addition of dasatinib to gemcitabine and docetaxel would be associated with even greater hematologic toxicity. A phase I evaluation would be required to determine if biologically relevant doses could be delivered with the triplet combination. However, the disparate sequential combination results in our cell lines was not expected and requires further study. Alternatively, doublet therapy with dasatinib plus gemcitabine or docetaxel may be more effective than triplet therapy.

We acknowledge the limitations of our study, which include the use of only two LMS cell lines rather than primary tumor, and the use of *in vitro* models. Further investigation in *in vivo* models is warranted to confirm our *in vitro* results. Further study is also needed to determine if dasatinib exerts its function via inhibition of the SRC pathway or via other tyrosine kinases, such as BCR-ABL, c-KIT, EPHA2, EGFR and PDGF, or a combination. In summary, we are the first to report an anti-proliferative effect of dasatinib in combination with cytotoxic agents in uterine leiomyosarcoma.

## Electronic supplementary material

Additional file 1: Figure S1: Expression of phospho SRC (pSRC) in the SK-UT-1 cell line. “KDR” are vascular endothelial growth factor cells use as control group. Compare to this group, both SKUT-1 and SKUT-1B demonstrated a much higher pSRC signal. (DOCX 15 KB)

Additional file 2: Figure S2: The total SRC protein expression after treatment with single agent dasatinib in SK-UT-1 and SK-UT-1B cell lines. In SK-UT-1, there was an increase in tSRC after treatment with single-agent dasatinib at 30 nm (148%, p<0.001), 100 nm (181%, p<0001) and 500 nm (172%, p<0.001) compared to controls. In SK-UT-1B, there was an increase in tSRC after treatment with single-agent dasatinib at 30 nm (152%, p<0.001), but a decrease at 100 nm (64%, p<0.001) and 500 nm (74%, p<0.001). (DOCX 16 KB)

Additional file 3: Figure S3: pSRC protein expression after treatment with single agent dasatinib in SK-UT-1 and SK-UT-1B cell lines. In SK-UT-1, pSRC levels were significantly decreased after treatment with dasatinib at 30 nm (24%, p<0.001), 100 nm (14%, p<0.001) and 500 nm (3%, p<0.001). In SK-UT-1B, there was a decrease in pSRC levels after treatment with single-agent dasatinib at 30 nm (17%, p<0.001), 100 nm (7%, p<0.001) and 500 nm (4%, p<0.001). (DOCX 16 KB)

Below are the links to the authors’ original submitted files for images.

Authors’ original file for figure 1

Authors’ original file for figure 2

Authors’ original file for figure 3

Authors’ original file for figure 4

Authors’ original file for figure 5

Authors’ original file for figure 6

Authors’ original file for figure 7

Authors’ original file for figure 8

Authors’ original file for figure 9

Authors’ original file for figure 10
