# Supplementary material for: Dasatinib (BMS-35482) potentiates the activity of gemcitabine and docetaxel in uterine leiomyosarcoma cell lines
Source: Gynecol Oncol Res Pract. 2014 Sep 30;1:2. doi: 10.1186/2053-6844-1-2 (PMC4877815; doi:10.1186/2053-6844-1-2)
Supplement: Supplementary file 1 — Additional file 1: Figure S1: Expression of phospho SRC (pSRC) in the SK-UT-1 cell line. “KDR” are vascular endothelial growth factor cells use as control group. Compare to this group, both SKUT-1 and SKUT-1B demonstrated a much higher pSRC signal. (DOCX 15 KB) [file 40661_2014_2_MOESM1_ESM.docx]

**Figure S1.** Expression of phospho SRC (pSRC) in the SK-UT-1 cell line**.** “KDR” are vascular endothelial growth factor cells use as control group. Compare to this group, both SKUT-1 and SKUT-1B demonstrated a much higher pSRC signal.
