# Supplementary material for: Dasatinib (BMS-35482) potentiates the activity of gemcitabine and docetaxel in uterine leiomyosarcoma cell lines
Source: Gynecol Oncol Res Pract. 2014 Sep 30;1:2. doi: 10.1186/2053-6844-1-2 (PMC4877815; doi:10.1186/2053-6844-1-2)
Supplement: Supplementary file 2 — Additional file 2: Figure S2: The total SRC protein expression after treatment with single agent dasatinib in SK-UT-1 and SK-UT-1B cell lines. In SK-UT-1, there was an increase in tSRC after treatment with single-agent dasatinib at 30 nm (148%, p<0.001), 100 nm (181%, p<0001) and 500 nm (172%, p<0.001) compared to controls. In SK-UT-1B, there was an increase in tSRC after treatment with single-agent dasatinib at 30 nm (152%, p<0.001), but a decrease at 100 nm (64%, p<0.001) and 500 nm (74%, p<0.001). (DOCX 16 KB) [file 40661_2014_2_MOESM2_ESM.docx]

**Figure S2.** The total SRC protein expression after treatment with single agent dasatinib in SK-UT-1 and SK-UT-1B cell lines. In SK-UT-1, there was an increase in tSRC after treatment with single-agent dasatinib at 30 nm (148%, p<0.001), 100 nm (181%, p<0001) and 500 nm (172%, p<0.001) compared to controls. In SK-UT-1B, there was an increase in tSRC after treatment with single-agent dasatinib at 30 nm (152%, p<0.001), but a decrease at 100 nm (64%, p<0.001) and 500 nm (74%, p<0.001).
