# Supplementary material for: Dasatinib (BMS-35482) potentiates the activity of gemcitabine and docetaxel in uterine leiomyosarcoma cell lines
Source: Gynecol Oncol Res Pract. 2014 Sep 30;1:2. doi: 10.1186/2053-6844-1-2 (PMC4877815; doi:10.1186/2053-6844-1-2)
Supplement: Supplementary file 3 — Additional file 3: Figure S3: pSRC protein expression after treatment with single agent dasatinib in SK-UT-1 and SK-UT-1B cell lines. In SK-UT-1, pSRC levels were significantly decreased after treatment with dasatinib at 30 nm (24%, p<0.001), 100 nm (14%, p<0.001) and 500 nm (3%, p<0.001). In SK-UT-1B, there was a decrease in pSRC levels after treatment with single-agent dasatinib at 30 nm (17%, p<0.001), 100 nm (7%, p<0.001) and 500 nm (4%, p<0.001). (DOCX 16 KB) [file 40661_2014_2_MOESM3_ESM.docx]

**Figure S3.**  pSRC protein expression after treatment with single agent dasatinib in SK-UT-1 and SK-UT-1B cell lines. In SK-UT-1, pSRC levels were significantly decreased after treatment with dasatinib at 30 nm (24%, p<0.001), 100 nm (14%, p<0.001) and 500 nm (3%, p<0.001). In SK-UT-1B, there was a decrease in pSRC levels after treatment with single-agent dasatinib at 30 nm (17%, p<0.001), 100 nm (7%, p<0.001) and 500 nm (4%, p<0.001).
