# Supplementary figures and images for: Dasatinib (BMS-35482) potentiates the activity of gemcitabine and docetaxel in uterine leiomyosarcoma cell lines
Source: Gynecol Oncol Res Pract. 2014 Sep 30;1:2. doi: 10.1186/2053-6844-1-2 (PMC4877815; doi:10.1186/2053-6844-1-2)

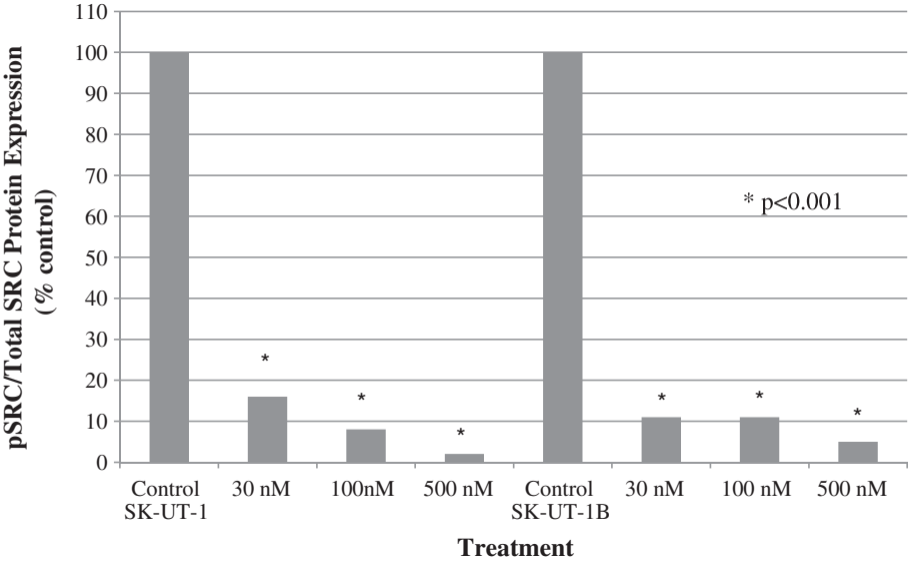

Supplement: Supplementary file 4 — Authors’ original file for figure 1 [file 40661_2014_2_MOESM4_ESM.pdf]

**p-paxillin/Total paxillin Protein Expression**

**(% of control)**

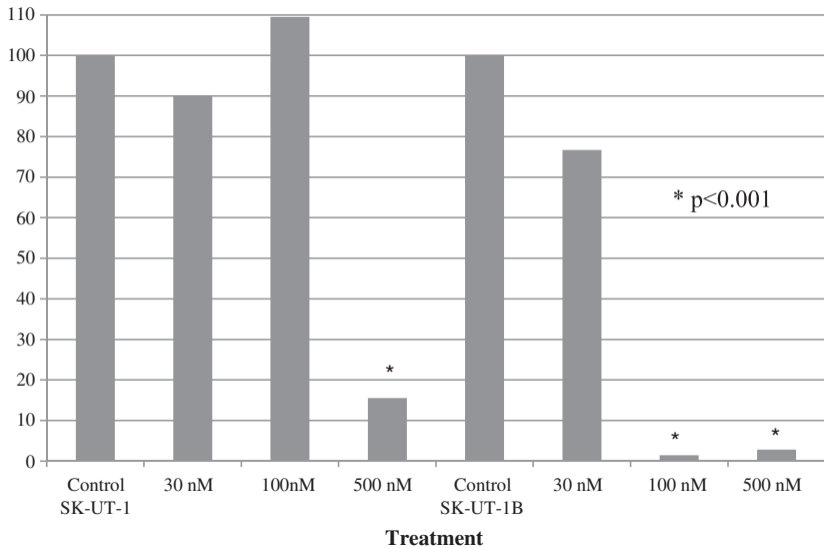

Supplement: Supplementary file 5 — Authors’ original file for figure 2 [file 40661_2014_2_MOESM5_ESM.pdf]

**SK-UT-1****SK-UT-1B**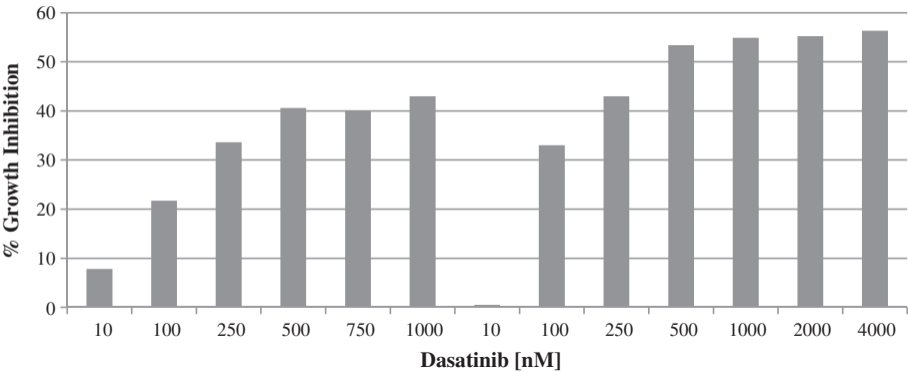

Supplement: Supplementary file 6 — Authors’ original file for figure 3 [file 40661_2014_2_MOESM6_ESM.pdf]

**A.****SK-UT-1**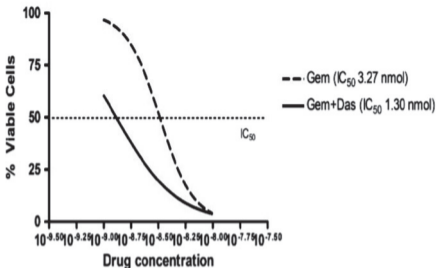**B.****SK-UT-1**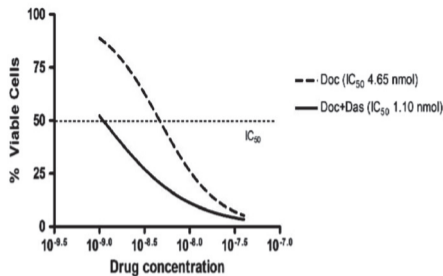**C.****SK-UT-1B**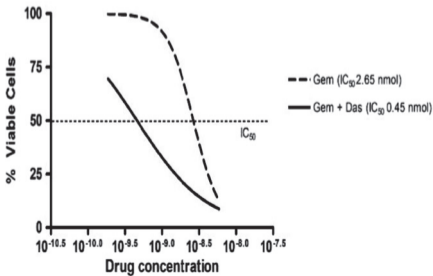**D.****SK-UT-1B**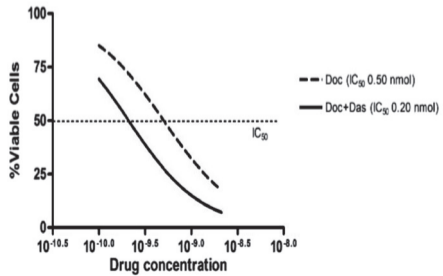

Supplement: Supplementary file 7 — Authors’ original file for figure 4 [file 40661_2014_2_MOESM7_ESM.pdf]

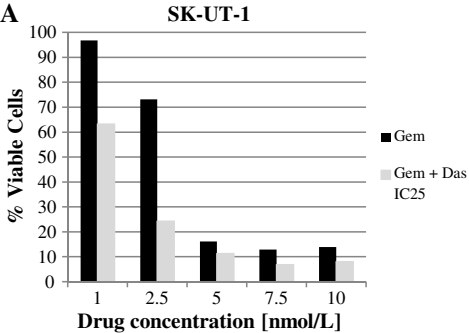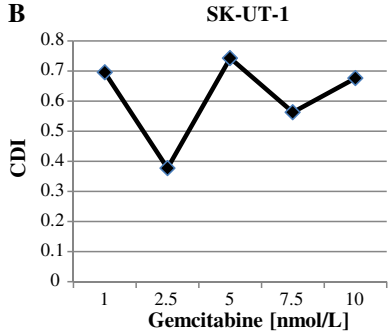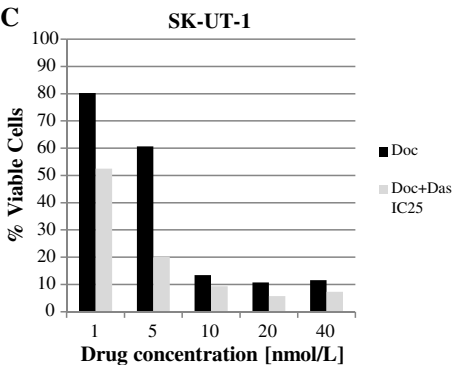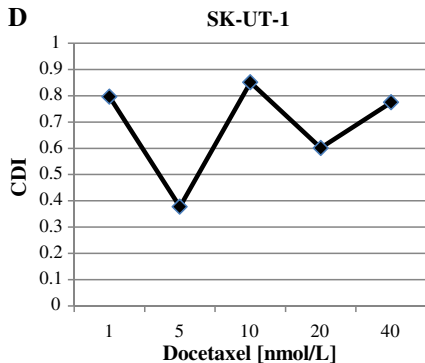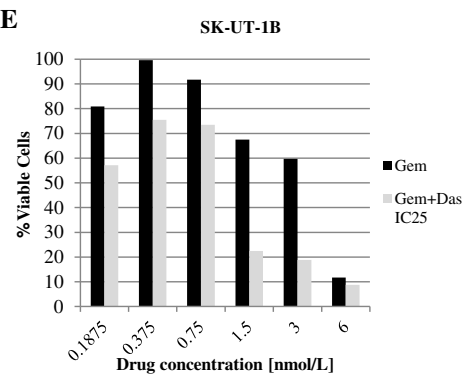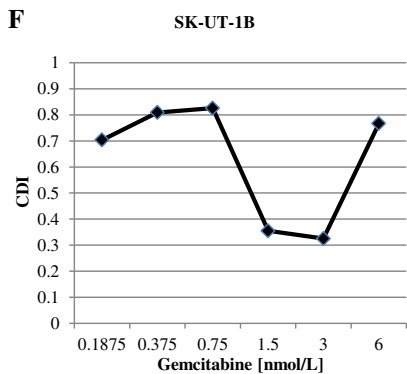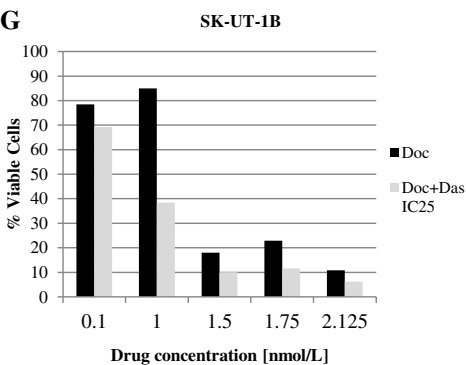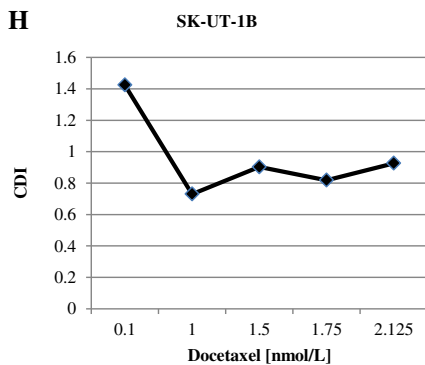

Supplement: Supplementary file 8 — Authors’ original file for figure 5 [file 40661_2014_2_MOESM8_ESM.pdf]
